# Anticorrosion Performance of PVDF Membranes Modified by Blending PTFE Nanoemulsion and Prepared through Usual Non-Solvent-Induced Phase Inversion Method

**DOI:** 10.3390/membranes11060420

**Published:** 2021-05-31

**Authors:** Tianshu Liu, Xiaoji Zhou, Yizhuo Sun, Renbi Bai

**Affiliations:** 1 Center for Separation and Purification Materials & Technologies, Suzhou University of Science and Technology, Suzhou 215009, China; liutianshu2021@163.com (T.L.); zhou-xiaoji@163.com (X.Z.); sunyizhuo2019@163.com (Y.S.); 2 School of Environmental Science and Engineering, Suzhou University of Science and Technology, Suzhou 215009, China; 3 Jiangsu Collaborative Innovation Center for Technology and Material of Water Treatment, Suzhou 215009, China

**Keywords:** PVDF/PTFE composite membrane, PTFE nanoparticle, polymer blending, non-solvent induced phase separation method, anticorrosive performance

## Abstract

In this study, PVDF/PTFE composite membranes were prepared by adding a PTFE nanoemulsion to a PVDF solution and casting it through the conventional non-solvent-induced phase separation method. The objective was to explore the effectiveness of using a simple and economical method to modify PVDF membranes with PTFE to enhance their anticorrosion performance, especially under highly acidic or alkaline conditions. PTFE nanoparticles (of around 200 nm in size) in nanoemulsion form were blended with PVDF at a mass ratio of PTFE:PVDF in the range of 0–0.3:1. The obtained membranes were examined to determine the effect of the added PTFE nanoparticles on the structure of the modified PVDF membranes as well as on their mechanical strength and surface characteristics. The membranes were then immersed in various concentrations of acidic or alkaline solutions for varied durations ranging from a few days up to as long as 180 days (6 months). The impacts of by the corrosive solutions on the tensile strength, surface roughness, and water flux of the membranes with different exposure times were quantified. The results showed that although a certain extent of change may occur with extended immersion times, greatly enhanced anticorrosion performance was obtained with the prepared PVDF/PTFE membranes compared with the unmodified PVDF membrane. For example, after being immersed in 5 mol-H^+·^·L^−^^1^ H_2_SO_4_, HCl, and HNO_3_ solutions for 6 months, the tensile strength at breaking point remained at up to 69.70, 74.07, and 71.38%, respectively, of the initial strength for the PVDF/PTFE (M30) membrane. This was in contrast to values of only 55.77, 70.43, and 61.78% for the unmodified PVDF membrane (M0). Although the water flux and surface roughness showed a change trends to the tensile strength, the PVDF/PTFE (M30) membrane had much higher stability than the PVDF (M0) membrane. In a continuous filtration experiment containing H_2_SO_4_ at 0.01 mol-H^+^·L^−1^ for 336 h (14 days), the PVDF/PTFE membrane showed a maximum flux change of less than 30%. This was in comparison with a change of up to 50% for the PVDF membrane. However, the PVDF/PTFE membranes did not seem to have a greatly enhanced anticorrosion performance in the alkaline solution environment tested.

## 1. Introduction

Many industries, including electroplating, semiconductor fabrication, acid mining, pharmaceutical manufacturing, and phosphorus acid production industries, often produce highly corrosive wastewater during their processes. This includes concentrated acidic or alkaline effluents that must be treated or purified to specific standards before discharge or reuse [1]. Currently, the methods used for treating corrosive wastewater, especially acidic or alkaline water, include neutralization, chemical precipitation, solvent extraction, membrane filtration, ion exchange, and adsorption [2,3,4,5,6]. Many of the conventional treatment methods still heavily rely on the use of expensive chemicals. Due to the highly corrosive or toxic nature of industrial effluents, the ability to treat them directly by a more economic biological method has been limited [4,5,6,7]. In recent years, membrane separation technology, a highly effective physical process, has increasingly and more widely been used for the treatment of various municipal and industrial wastewaters due to its many social, environmental, and economic benefits, for example, its high effluent quality, the potential for resource recovery and reuse, the reduction or elimination of secondary pollution, and the low carbon emissions. The interest in membrane technology for application in the treatment of highly corrosive industrial effluents, particularly for the recovery of acids and metal components and the reuse of treated effluents to attain a zero discharge status, has increased considerably over the years [8,9]. Generally, when effluents are only mildly corrosive, many conventional membranes that are available in the market can be satisfactorily used to purify wastewater. However, there have been many situations where the industrial wastewater generated, for example, in the metal plating and acid mining industries, can have a very high acid concentration and, therefore, be highly corrosive. Most of the conventional or available membranes in the market are not tolerated that well, and the membranes have often been limited to a pH application range of 2–12 or even narrower.

The two types of membranes that may currently be used for highly corrosive industrial wastewater treatment include ceramic membranes and polytetrafluoroethylene (PTFE) membranes [10,11]. Ceramic membranes, which are mainly made of metal oxides and are very stable, are generally expensive with high production costs and high operational energy consumption and often require stringent installation conditions, as they are brittle with low elasticity, all of which greatly limits their application as the first choice in many situations [12,13]. The C-C bonds of the skeleton of PTFE, a unique organic polymer, are perfectly protected from chemical degradation due to its specially formed molecular structure (the strong electronegativity of the F atom and the complete helical sheath structure formed by the electron cloud of the C-F covalent bonds). Therefore, PTFE membranes show excellent corrosion resistance or chemical inertness [14]. Nowadays, PTFE membranes are increasingly being used in various applications, such as exhaust-gas treatment, membrane distillation (MD), and oil–water separation [15,16,17,18]. Zhu et al. [17] prepared hollow fiber PTFE membranes through a cold-pressing method and achieved a salt rejection rate of 99.9%. Xue et al. [18] used catechol (CA) and polyethyleneimine (PEI) to hydrophilically modify flat PTFE membranes for oil–water separation. The as-prepared hydrophilized flat PTFE membranes have excellent oil resistance with an oil rejection ratio of near 99%. Although PTFE membranes, as a type of organic membrane, may have greater flexibility in regard to fabrication into various forms, they cannot be manufactured by the more economical and well-established non-solvent-induced phase separation (NIPS) method [19]. The cost of PTFE materials is several times higher than that of PVDF materials, and the more costly thermal preparation process for PTFE membranes has made them much more expensive than other organic polymeric membranes on the market. Furthermore, solo PTFE membranes display a superhydrophobic feature and have very poor water permeability as well as a low mechanical strength [20]. These features have made solo PTFE membranes less viable for use in industrial wastewater treatment, especially in large-scale practical applications.

One of the most popular organic membranes used in practical applications is polyvinylidene fluoride (PVDF) membranes. They show moderate acid and alkali resistance (in the pH range of 2–12) and chemical stability and are used for the treatment of many commonly found industrial wastewaters. PVDF has a similar spatial configuration and constituent elements to PTFE and, therefore, shares some of its advantages. However, PVDF, which is much cheaper than PTFE, can be easily dissolved in many popular organic solvents, such as DMF, DMSO, and NMP, and the membranes can be prepared by the traditional and well-established NIPS method at a greatly reduced production cost. PVDF membranes have widely been used for water and wastewater treatment, and they have a widely accepted system design and operational experience. Since, unlike PTFE membranes, PVDF membranes are not highly tolerant to corrosive industrial wastewaters, such as wastewater with a pH lower than 2 or high than 12, it has been of great research and engineering interest to improve or enhance the anticorrosive property of PVDF membranes to allow their application to be expanded, for example, into acidic or alkaline industrial wastewater treatment.

One approach is to prepare PVDF/PTFE composite membranes [21,22,23,24,25]. Most approaches of this type have to use the more expensive method of thermally induced phase inversion (TIPI). This usually involves heating materials to up to 220 °C or above, because PTFE has no known solvent able to obtain the PVDF/PTFE membrane casting solution [23]. There have also been some attempts to prepare PVDF/PTFE composite membranes through the common and less-energy-intensive NIPS method by blending PTFE powder with PVDF. It has been reported that the addition of 4 μm of PTFE powder to PVDF in the 0 to 12 wt% range can be used to prepare nanofibrous PVDF-PTFE membranes through electrostatic spinning [24]. This was shown to produce membranes with a water contact angle (WCA) in the range of 130.4°to 152.2° and an increase in the liquid entry pressure (LEPw) of the pore of the membranes from 84 to 137 kPa due to the greatly increased hydrophobicity. The electrostatic spinning method requires a high voltage of up to 30 KV, which may limit its large-scale industrial application. In membrane distillation, Teo et al. [25] prepared a PVDF/PTFE membrane by dispersing PTFE particles (<1 μm) into a 20–40 wt% PVDF cast solution and fabricating the membranes using a dry-jet wet-spinning process. They found that the addition of PTFE microparticles greatly suppressed the formation of macrovoids and increased the surface hydrophobicity of the obtained membranes. The prepared membrane became a good candidate for membrane distillation rather than for the permeation of water and wastewater in the normal membrane filtration process. Khumalo et al. [22] prepared PVDF/PTFE/fMSNs nanocomposite membranes using the phase inversion technique through the immersion precipitation method by inserting a polymer solution cast on a glass plate into a deionized water coagulation bath. The increase in PTFE loading improved the membrane structure, resulting in the formation of smaller, evenly distributed pores and a porous, spongy structure, which could be used in MD to recover water from hydrolyzed human urine. The PTFE particles used in previous reports were usually relatively large at the micrometer level (average particle size of around 1–5 µm). It was also found that the agglomeration of these particles inevitably occurred in the membrane casting solution, which also affected the uniformity of the formed membrane’s structure [26,27]. There has also been a lack of information on how to improve the surface and internal structure of PVDF membranes by blending them with PTFE particles to enhance the anticorrosion performance. 

In this study, we examined the modification of PVDF membranes by blending PTFE nanoparticles. PTFE nanoparticles with an average size of around 220 nm were dispersed in a liquid emulsion. The PTFE nanoemulsion was added to the PVDF/NMP dope system in certain specific ratios to obtain a uniform casting solution. Then, PVDF/PTFE composite membranes were prepared by the traditional and low-cost common NIPS method instead of the higher-cost thermal preparation method. Compared with previous studies that used PTFE microparticles (powder), the aggregation of PTFE nanoparticles was greatly suppressed, and PTFE nanoparticles were dispersed more uniformly in the PVDF matrix. The morphology, mechanical properties, water flux, and rejection performance of the PVDF/PTFE composite membranes were investigated before and after their immersion in corrosive solutions, including strong acidic or alkaline solutions, for varied time periods of up to 6 months. Our objective was to examine the modification of PVDF membranes by blending PTFE nanoparticles in a nanoemulsion form, prepared with the conventional non-solvent-induced phase inversion method, and investigate a possible enhancement in the anticorrosion performance of the obtained PVDF/PTFE composite membranes. Our aim was to expand the application of PVDF membranes into the treatment of more challenging industrial wastewaters with strong acidity or alkalinity.

## 2. Experimental Section

### 2.1. Materials

PVDF powder and PTFE nanoemulsion containing 62 wt% PTFE nanoparticles with an average size of 220 nm, dispersed in water with a nonionic surfactant as a stabilizer, were obtained from Shanghai 3F New Material Technology Co. Ltd., Shanghai, China N-methyl pyrrolidone (NMP) was provided by Tianjin Zhiyuan Chemical Reagent Co., Ltd., Tianjin, China. Sulfuric acid (H_2_SO_4_), nitric acid (HNO_3_), hydrochloric acid (HCl), anhydrous sodium dihydrogen phosphate (NaH_2_PO_4_), and anhydrous disodium hydrogen phosphate (Na_2_HPO_4_) were supplied by Macklin. Sodium hydroxide (NaOH) and bovine serum albumin (BSA) were purchased from Aladdin, and n-butanol and sodium dodecyl sulfate (SDS) were purchased from Titan Technology Co. Ltd., Shanghai, China.

### 2.2. Preparation of PVDF/PTFE Composite Membrane

The PVDF powder was first dried overnight at 60 °C and then cooled to room temperature naturally. A specific amount of dried PVDF powder was added to NMP at 70 °C with stirring to obtain a uniform and transparent PVDF casting solution. Then, a different amount of the PTFE nanoemulsion was added dropwise as needed to the PVDF casting solution, and the mixture was stirred continuously for another 2 h at the same temperature for homogenization. Finally, the obtained PVDF/PTFE solution was allowed to stand at 70 °C for 24 h for the removal of any air bubbles that were possibly entrapped in the solution. The degassed PVDF/PTFE solution was then carefully distributed on a glass plate and stretched into a uniform membrane by a membrane scraper (Elcometer 4340, Elcometer) at 25 °C. The PVDF/PTFE composite membrane was formed by the common NIPS method, with the film on the glass plate being left in the air for 30 s and then subsequently immersed into a coagulated bath with deionized water at 45 °C for 2 h. The formed membrane film was then transferred to another deionized water bath at room temperature for another 3 days, during which the water was replaced by fresh water on a daily basis. Finally, the obtained membrane was taken out and naturally dried on a shelf before being stored for characterization analysis or other tests. Several PVDF/PTFE composite membranes with different PVDF:PTFE ratios were prepared, and their basic information is given in Table 1. 

### 2.3. Chemical Stability Test Conducted on the Prepared Membranes

A series of H_2_SO_4_, HCl, and HNO_3_ solutions with an H^+^ concentration of up to 5 mol·L^−1^ (pH << 0) and a NaOH solution with an OH^−^ concentration of 0.1 mol·L^−1^ (pH = 13) were prepared. The chemical stability of the prepared membranes was tested by immersing the membrane samples into the abovementioned acidic or alkaline solutions for a varied amount of time of up to 180 days (6 months). At different durations, the membrane samples were taken out and repeatedly washed with deionized water to remove the acid or alkaline solution on the membrane surface. Then, the membrane was immersed in a deionized water bath for at least 1 day, and the water was replaced by freshwater regularly until its pH was neutral. Finally, the sample was dried at room temperature. The samples were subsequently analyzed to determine their characteristics or performance features, including examination of the surface structure, mechanical strength, and water flux. For simplicity, we used the letters “S”, “Cl”, “N”, and “Na” as subscripts to represent membranes immersed in H_2_SO_4_, HCl, HNO_3_, and NaOH solutions, respectively. The number following these subscript letters was used to indicate the duration of immersion in the corresponding solution (in months). For example, M0 immersed in H_2_SO_4_ solution for 1 month (30 days) is denoted as M0_S1_.

### 2.4. Characterization of Membranes

#### 2.4.1. Microimage Analysis

The surface and cross-sectional morphologies of the various prepared membranes were examined through scanning electron microscopy (SEM, Quanta FEG250, FEI Company). Prior to the analysis, samples were dried under vacuum and coated with a gold layer according to the specific requirements of the equipment operation. To maintain an intact cross-section of the sample for analysis, a strip of the membrane sample to be scanned was frozen and fractured in liquid nitrogen immediately prior to sample preparation for the SEM scan.

The surface morphology or roughness of the various membranes before and after the corrosion test was also examined with an atomic force microscope (AFM, Multiomode 8, Bruker). The Nanoscope Version 1.9 software and the AFM were used for image acquisition. The AFM images were captured as a 3D model, and the measurement area had dimensions of 5 µm × 5 µm.

#### 2.4.2. Mechanical Properties

The mechanical properties of the prepared membranes were estimated before and after the corrosion treatment by measuring the tensile strength and elongation at break using Instron equipment (Model 5944 tensile analyzer). A 7 cm × 1 cm strip of a membrane sample was prepared, and then the 2 ends were attached to the 2 fixtures of the instrument with a separation distance of 5 cm. The 2 fixtures moved away slowly at a velocity of 10 mm·min^−1^ until the membrane fractured, and the tensile strength and elongation at break were recorded automatically by the instrument. A total of 6 measurements were made for each membrane sample, and the average results were reported.

#### 2.4.3. Water Contact Angle Measurements

WCA measurements were performed to evaluate the effect of blending the PTFE nanoemulsion with PVDF on the surface hydrophilicity/hydrophobicity of the PVDF/PTFE composite membranes. The measurements were performed with the sessile drop method using a Rame-hart goniometer (Ramé-hart 500). A dried 1 cm × 3 cm membrane sample was attached to the sample slide of the goniometer. Then, 3 µL of deionized water was dropped onto the surface of the membrane sample using a microsyringe. The instrument was set to immediately record an image of the water droplet and its changes and then determine the contact angle between the membrane surface and the water droplet/air interface automatically. All measurements were carried out at room temperature (25 °C). The measurements were made at 15 randomly selected locations on the membrane surfaces, and the average WCA from these measurements was calculated and reported as the representative WCA for the measured membrane sample.

#### 2.4.4. Membrane Porosity

The porosity of the prepared membranes was estimated by the liquid wetting method, as reported in the literature [28]. A membrane sample with a measured area and thickness that was vacuum-dried at 60 °C was first immersed completely in 500 mL of n-butanol for 12 h at 25 °C to allow the membrane sample to be completely wetted. Then, the wetted membrane was taken out and the n-butanol was wiped off the surface. The wetted membrane sample was immediately weighed, and the weight was determined in m_2_ (g) with an electrical analytical balance. The wet membrane sample was then vacuum-dried at 60 °C (VD115, Binder) to a constant weight. Its weight was recorded in m_1_ (g). The approximate porosity of the measured membrane sample was estimated using Equation (1) as follows:

(1) $$P=\frac{m_{2}-m_{1}}{\rho \cdot A\cdot h}\times 100\%$$

where “P” is the porosity of the membrane (%), “*ρ*” is the density of n-butanol (g·cm^−3^), “A” is the area of the measured membrane sample (cm^2^), and “h” is the thickness of the membrane sample (cm) determined from the SEM analysis.

#### 2.4.5. Membrane Permeation Test

A filtration cell made of a pressure controller, a clear water reservoir, and cell stirred with a magnetic stirrer (MSC050, Shanghai Mosu Science Equipment Co. Ltd., Shanghai, China) was used in the laboratory to evaluate the water permeation properties of the obtained membranes before and after their immersion in a corrosive solution. A circular slice of the membrane with an effective area of 10.17 cm^2^ was installed in the stirred cell. Initially, the membrane slice was pre-compacted for 0.5 h at 0.15 MPa with deionized water. Then, the membrane cell was fed with deionized water at 0.1 MPa, and the water flux through the membrane was measured and calculated using Equation (2):

(2) $$J=\frac{\Delta V}{S\cdot \Delta t}$$

where J denotes the pure water flux (L·m^−2^·h^−1^), ΔV is the amount of permeate (L) collected through the membrane within the filtration period Δt (h), and S is the effective filtration area of the membrane (m^2^), respectively. For each membrane, the measurement was repeated thrice, with the average result being reported in this study.

An identical device was used to filter the H_2_SO_4_ solution with an H^+^ concentration of 0.01 mol·L^−1^ for as long as 336 h (14 days), and the fluxes were monitored at various time intervals. The fluxes were also calculated using Equation (2).

## 3. Results and Discussion

### 3.1. Membrane Morphologies and PTFE Dispersion

**Figure 1:**
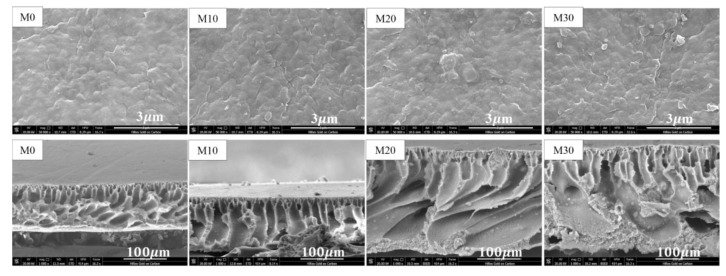
Top surface (**up**) and cross-sectional (**down**) SEM images of the prepared membranes.

The morphologies of the surface and cross-section of the prepared PVDF/PTFE membranes measured in the SEM analyses are presented in Figure 1. The blending of PTFE did not appear to have a very noticeable effect on the surface and cross-section structures of the membranes, although it did greatly impact the thickness of the membranes. All membranes had a dense skin layer supported by a more porous sublayer with finger-like voids. However, as the amount of PTFE nanoparticles blended in PVDF increased, the porosity of the prepared membranes increased greatly from 73.9% for membrane M0 to 80.3% for membrane M30 (see Table 1). The higher porosity of the membranes with PTFE nanoparticles was probably caused by two factors: (1) The liquid in the PTFE nanoemulsion, which was added with the PTFE nanoparticles into the PVDF/PTFE composite, and may have behaved as a porogen and increased the pore voids, contributing to a greater porosity in the obtained composite membrane; and (2) the compatibility between PVDF and PTFE nanoparticles was lower in PVDF/PTFE composite membranes (i.e., M10–M30), which may have led to a greater amount of liquid being retained in the membrane structure, leading to the final membrane having greater porosity than the PVDF in the solo PVDF membrane (M0). The cross-sectional images of the PVDF/PTFE composite membranes show larger solid particles or aggregates, especially for membranes with a higher amount of PTFE (e.g., M30), indicating the possibility of some extent of aggregation in the added PTFE nanoparticles. This situation was, however, less evident for the M10 membrane, suggesting that adequate blending of PTFE nanoparticles with PVDF was achieved at a lower ratio of 10%. It is interesting to note that the PTFE nanoparticles appeared to be more concentrated at the membrane surface, rather than being distributed more uniformly to the entire structure of the PVDF/PTFE composite membranes, as occurs under the current NIPS preparation method. This phenomenon could improve the anticorrosion performance of the prepared membranes because more PTFE particles appear on the surface, and they have much greater corrosive resistance than PVDF. Another implication of this phenomenon was that, by changing the composition of the nonsolvent used in the NIPS method, it may be possible to control how the PTFE nanoparticles are distributed within the structure of the prepared membranes and thus control the surface property of the membranes being prepared.

**Figure 2:**
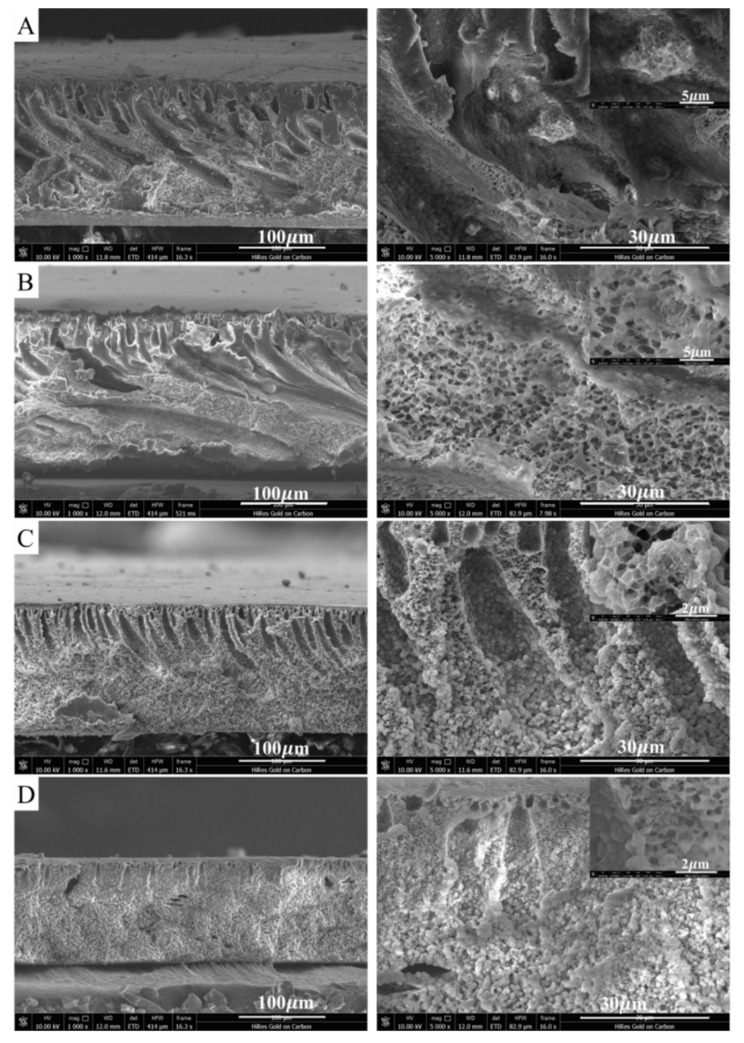
Agglomeration phenomenon of micro-PTFE particles and nano PTFE particles in the PVDF/PTFE composite membrane (PTFE was about 10% of PVDF in mass). (**A**): the M_micro_ membrane blended with 5 μm PTFE micro particles; (**B**–**D**): the membrane blended with PTFE nanoemulsion with 0%, 4%, and 8% SDS added, respectively.

To compare the aggregation phenomenon that has been reported to occur when blending PTFE nanoparticles with PTFE microparticles in some earlier studies, we also prepared a PVDF/PTFE composite membrane named M_micro_ by blending 10% PTFE microparticles with an average size of 5 μm in the same way as was conducted for the M10 membrane. As shown in Figure 2A, the SEM images indicated that the PTFE microparticles formed larger agglomerates in the size range of around 7–10 μm to those formed in the NIPS process, and the aggregates were observed to be randomly distributed inside the membrane’s cross-sectional structure. On the contrary, the blended PTFE nanoparticles seemed to be more uniformly dispersed in the membrane’s structure, and although agglomeration occurred, the agglomerates formed had a much smaller size of <5 μm.

To explore the possibility of further improving the dispersion of PTFE nanoparticles in the PVDF/PTFE composite solution, we tested the addition of different amounts of SDS surfactant to the PVDF/PTFE casting solution. This anionic surfactant was expected to change the surface tension of the PTFE nanoparticles and, thus, improve their stability and avoid their aggregation during the NIPS process [29]. Although the addition of SDS surfactant may be expected to reduce the aggregation of PTFE nanoparticles, experiments indicated that the content of SDS surfactant needed to be controlled at a maximum solubility of no greater than 10% to achieve an adequate solution. In Figure 2, we present SEM images of the cross-sections of PVDF/PTFE composite membranes prepared with 0%, 4%, and 8% SDS, respectively, that were added to the cast solution. It was found that the addition of SDS indeed improved the dispersion of the PTFE nanoparticles and greatly reduced the aggregation phenomenon, with the largest agglomerate measured as being around 1.35 µm for the membrane with 8% SDS. In addition, with an increase in the SDS content, the cross-sectional structure of the membranes changed significantly to have fewer macropores and a considerable increase in the sponge layer thickness. The finger-like voids almost disappeared completely for the PVDF/PTFE membranes with 8% SDS (see Figure 2D). The lower agglomeration and more uniform structure of the PVDF/PTFE composite membranes indicated better dispersion of the PTFE nanoparticles and perhaps better anticorrosion performance for the PVDF/PTFE composite membranes.

### 3.2. Membrane Strength and Surface Properties

The measured mechanical properties of the prepared membranes are also given in Table 1. In terms of the tensile strength, the solo PVDF membrane, M0, showed the highest value of 4.16 MPa. With the addition of PTFE nanoemulsion, the tensile strength of the PVDF/PTFE composite membranes appeared to reduce: 3.74 MPa for M10, 3.25 MPa for M20, and 2.97 MPa for M30, respectively. The breaking elongation also decreased as the PTFE nanoemulsion content increased: 246.8% for M0, 217.2% for M10, 175.2% for M20, and 131.0% for M30, respectively. The results indicate that the lower compatibility between PVDF and PTFE decreased the strength of the PVDF/PTFE composite membranes to a certain extent as compared with the solo PVDF membrane. Nevertheless, as intended for MF or UF membranes, the tensile strength of the PVDF/PTFE composite membranes was generally acceptable [30], especially for the PVDF/PTFE composite membrane M10, whose strength was very close to that of M0.

PTFE material is extremely hydrophobic, with literature reported WCA values exceeding 150° [24]. The addition of PTFE nanoparticles to PVDF was indeed found to increase the WCA of the prepared PVDF/PTFE composite membranes, with 78.6° for M0 in comparison with 81.2°, 83.8°, and 88.5° for M10, M20, and M30, respectively, although the impact on the membranes’ hydrophilicity was not significant. The oil contact angle of the membranes in water was also measured by the captive bubble method [31]. As also given in Table 1, the underwater oil contact angles were as follows: 100.3° for M0, 106.1° for M10, 114.7° for M20, and 125.0° for M30. The composite membranes were able to achieve significantly higher oil contact angles in water compared with the pure PVDF membrane (M0) as the concentration of blended PTFE nanoparticles increased. A higher oil contact angle indicates greater membrane anti-fouling performance, allowing the membrane to resist foulants such as organic pollutants, protein, and oil. The enhanced oleophobic performance of the PVDF/PTFE composite membranes can be attributed to the oleophobic component of the PTFE nanoparticles on the surface and cross-section of the prepared membranes.

### 3.3. Anticorrosion Stability of PVDF/PTFE Composite Membranes

The performance of the prepared membranes was monitored for 180 days to determine their durability by immersing them in H_2_SO_4_, HNO_3_, or HCl acid solutions with a [H^+^] concentration of 5 mol·L^−1^ and in a NaOH solution with an [OH^−^] concentration of 0.1 mol·L^−1^. At different immersion times, the membrane samples were taken out for the analysis of various characteristics, including mechanical strength, surface roughness, and water permeability, to evaluate the stability of the membranes.

**Figure 3:**
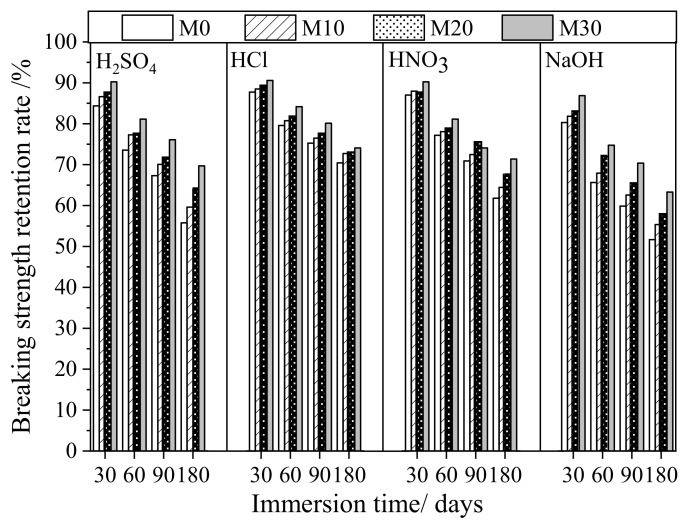
Rate of retention in tensile strength over time for prepared membranes immersed in acid or alkaline solutions.

Figure 3 and Table 2 present the variation in the tensile strength of the different types of membranes in the corrosion experiments. While the strength gradually weakened for all membranes as the immersion time increased from 30 to 180 days, the breaking strength retention rate of the M0 membrane immersed in the H_2_SO_4_ solution for 6 months was reduced to only 56%, whereas those for M10, M20, and M30 were reduced to 60%, 64%, and 70%, respectively. This indicates that the composite membranes containing PTFE nanoparticles were much more resistant to corrosion by the H_2_SO_4_ solution, having a lower reduction in the breaking strength. The results obtained for the HCl and HNO_3_ solutions were better than those obtained for the H_2_SO_4_ solution, and, at the same H^+^ concentration, the HCl solution was the least corrosive for the membranes. The breaking strength retention rate of membrane M0 immersed in the HCl and HNO_3_ solutions for 6 months was 70.4% and 61.8%, respectively, while for M30, it was 74.1% and 71.4%. It is evident that after blending PTFE nanoparticles with PVDF at a content of 10% to 30%, the prepared composite membranes were indeed more resistant to corrosion by typical acids than the PVDF membrane.

Both PVDF membranes and the PTFE/PVDF composite membranes showed slightly poorer anti-alkali corrosion properties when exposed to the 0.1 mol·L^−1^ NaOH solution. The breaking strength retention rate for the M0, M10, M20, and M30 membranes was 51.7%, 55.3%, 57.8%, and 63.3%, respectively, after corrosion in 0.1 mol·L^−1^ NaOH solution for 180 days (tested time was lower than those used for the acidic solutions). However, the PVDF/PTFE composite membranes (M10 to M30) again showed better resistance than the PVDF membrane (M0) to the alkaline solution tested.

The results presented in Figure 3 and Table 2 confirm that, compared with the pure PVDF membrane, the PVDF/PTFE composite membranes were more resistant to acidic and alkaline solutions. The greater the blending ratio of the PTFE nanoemulsion, the better the acid and alkali resistance of the prepared composite membranes. However, in consideration of the effect on the initial breaking strength of the composite membranes, different blending ratios of PTFE nanoparticles with PVDF may be chosen depending on the specific practical engineering applications.

**Figure 4:**
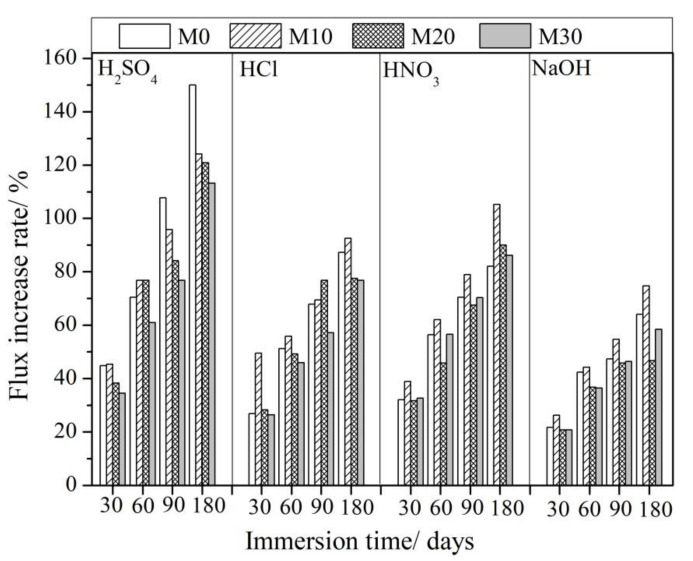
Schematic diagram of the change in water flux of the membrane before and after acid-base corrosion.

Figure 4 and Table 3 present the measured water flux changes for the different types of membranes before and after the immersion corrosion experiments. All membranes showed an increase in water flux after the corrosion tests, which indicates that the membrane pores or membrane hydrophilicity may have changed due to the acidic or alkaline treatment. The flux through the modified membranes appeared to be stable for a longer period of time under conditions of acidic corrosion, and its rate of increase was slower. For example, the flux change rate of the M30 membrane immersed in the acid solution for 6 months showed a similar change rate to the M0 membrane immersed for only 3 months in the same acidic solution. The flux of the pure PVDF membrane, M0, immersed in the H_2_SO_4_ solution for 3 months increased by 107.7% and continued to increase to 150.0% of the original value after 6 months. The membranes blended with PTFE nanoparticles, i.e., M10, M20, and M30, only had increases in water flux of 124.2%, 120.8%, and 113.2%, respectively, after being corroded in the same H_2_SO_4_ solution for 6 months.

The lower increase in flux after immersion in the NaOH solution as compared with the acidic solutions was mainly due to the lower molar concentration of the NaOH solution. The flux of M0 increased by 64% after immersion in the NaOH solution with an OH^−^ concentration of 0.1 mol·L^−1^ for six months. The increase in flux of the M10 membranes appeared to be greater, but that of M30 was reduced by about 58%. It seems that the membranes obtained by blending PTFE nanoparticles with PVDF may be more sensitive to alkaline solutions. Defluorination of PVDF polymers may occur in alkaline solutions, causing collapse or blockage of some membrane pores, causing the increase in flux to be minimal.

**Figure 5:**
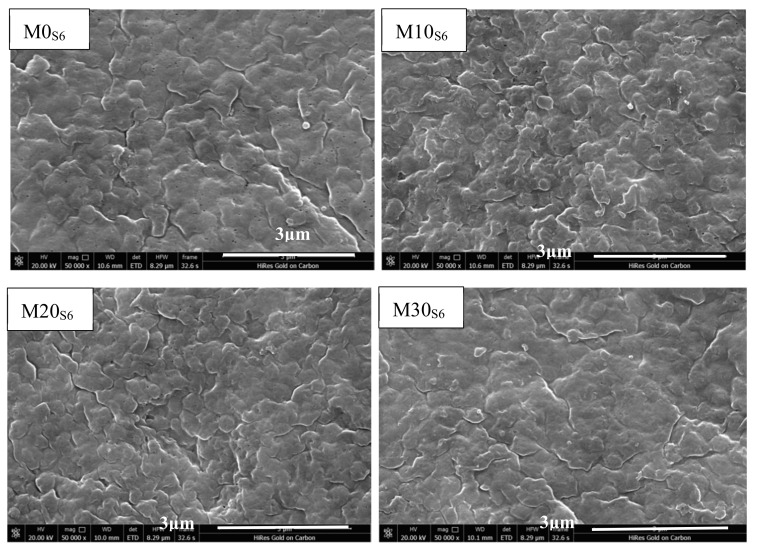
Surface SEM images of the prepared membranes after corrosion in the H_2_SO_4_ solution for 6 months.

The variation in the surface morphology of the prepared membranes before and after the corrosion tests provides information on the stability of the various membranes against acidic or alkaline conditions. SEM images of the surface morphologies of the prepared membranes after immersion in the H_2_SO_4_ solution for 6 months were obtained and are shown in Figure 5. The membrane surface was found to contain smaller pores than those that were present on the initial membranes (Figure 1). This may be mainly attributed to the hydrolysis caused by the catalytic oxidation of acid by the PVDF polymer and through the permeation and diffusion of polymer materials by corrosive media [32,33]. The number of pores, however, decreased obviously as the content of PTFE nanoparticles increased, as almost no obvious pores were observed on the surface of M30, which supports the hypothesis that the addition of PTFE nanoparticles to PVDF can improve or inhibit the corrosion of the composite membrane by acidic solutions.

**Figure 6:**
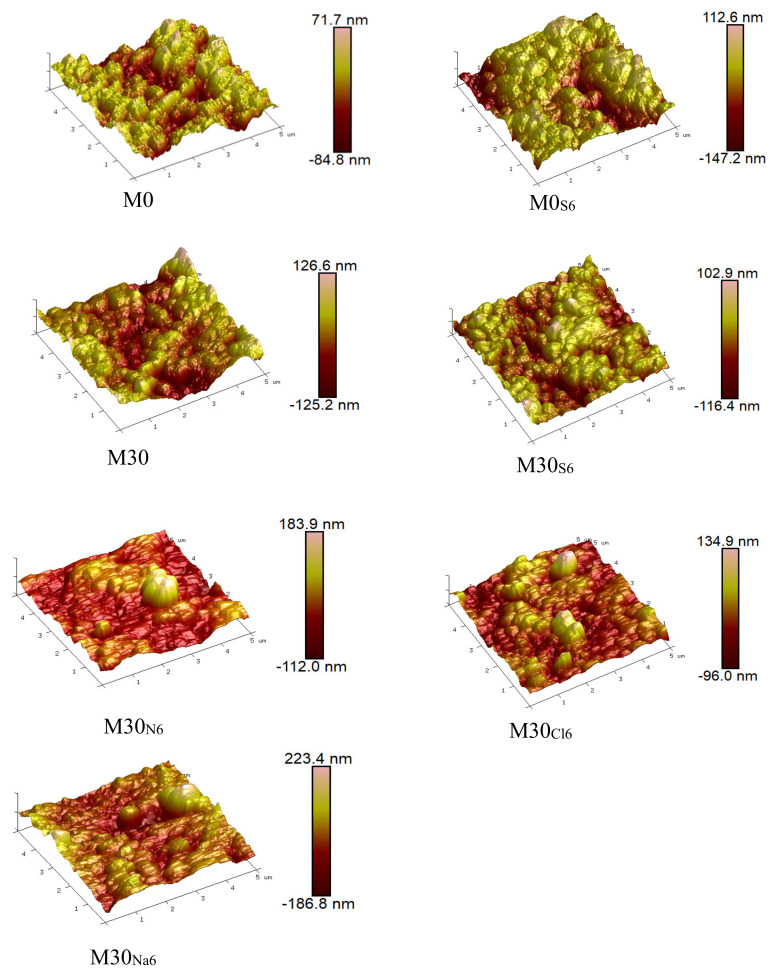
Three−dimensional AFM morphologies of the prepared membranes.

AFM was utilized to investigate the surface roughness of the membranes to provide evidence of the corrosion effect. Many studies have reported an increase in the roughness of used membranes after corrosion treatment [34,35]. In Figure 6 and Table 4, we present the AFM images and the measured average roughness of membrane M30 (as a representative), before and after corrosion in 5 mol·L^−1^ H_2_SO_4_, HCl, and HNO_3_ solutions and in 0.1 mol·L^−1^ NaOH solution for 6 months. The average roughness of PVDF membrane M0 was initially 15.7 nm, and that of the PVDF/PTFE composite membrane M30 was about 17.6 nm. The PTFE nanoparticles that migrated to the surface may have contributed to the slightly greater roughness of the M30 membrane. The average roughness of membrane M0 increased by 43%, from 15.7 to 22.5 nm, after immersion in the H_2_SO_4_ solution for 6 months. In comparison, the surface corrosion resistance of the prepared PVDF/PTFE composite membranes improved significantly with the addition of the PTFE nanoparticles. After immersion in H_2_SO_4_ solution (of the same concentration) for 6 months, the average roughness of membrane M30 only increased by 19%, from 17.6 nm to 21.0 nm. Compared with the H_2_SO_4_ and HNO_3_ solutions, which had similar corrosive effects on the membranes, the corrosion of the membranes by the HCl solution was obviously weaker. The average roughness of M30 increased from 17.6 to 18.5 nm after corrosion by the HCl solution for 6 months. While the composite PVDF/PTFE membranes showed greater resistance to a nonoxidizing acid (HCl), the membranes indeed suffered some oxidative degradation by strong oxidizing acids such as HNO_3_ and H_2_SO_4_. On the other hand, the membranes seemed to have poorer alkali resistance. The average roughness of membrane M30 increased by 25% and reached 22.0 nm after immersion in the NaOH solution for six months. This was higher than the roughness produced following immersion in the acidic solutions. PVDF is known to defluorinate when attacked by alkaline solution. Therefore, an alkaline solution can have a greater influence on the surface morphology and performance of PVDF-based membranes.

**Figure 7:**
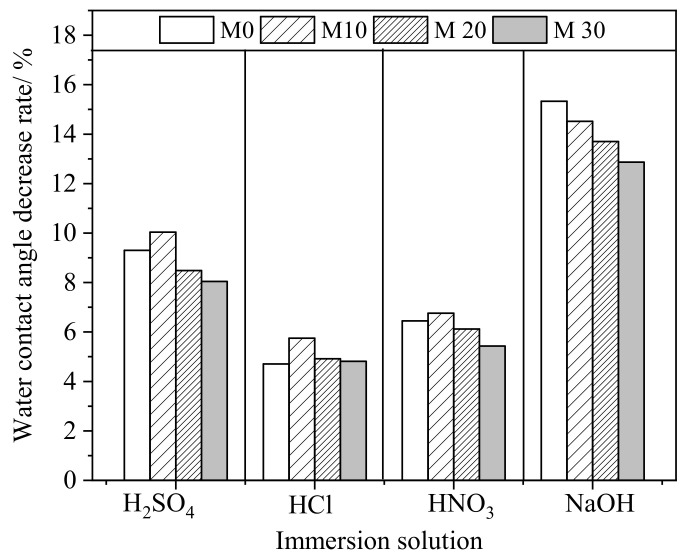
Rate of decrease in the water contact angle of the prepared membranes after corrosion in 5 mol·L^−1^ acid solutions and an 0.1 mol·L^−1^ alkaline solution for 6 months.

The change of water contact angle (WCA) may partly reflect the effect of membrane surface corrosion. The WCA values of the membranes before and after corrosion in 5 mol·L^−1^ H_2_SO_4_, HCl, and HNO_3_ solutions and 0.1 mol·L^−1^ NaOH solution for 6 months are presented in Figure 7 and Table 5. In general, the WCA of the membranes dropped to different degrees due to corrosion of the acidic or alkaline solution, becoming more hydrophilic. The water contact angle of membrane M0 decreased by 9.3%, 4.7%, or 6.4%, from an initial angle of 78.6° to 71.3°, 74.9°, and 73.5°, respectively, after immersion in H_2_SO_4_, HCl, or HNO_3_ solution for 6 months. Consistent with the previous analysis, a lower drop in the water contact angle occurred with the HCl solution as compared with stronger oxidizing acids such as HNO_3_ and H_2_SO_4_. The influence of 0.1 mol·L^−1^ NaOH on the WCA was much greater than with other solutions, and the WCA of membrane M0 decreased by 15.3%, from 78.6^o^ to 66.6^o^. The decrease in the WCA after corrosion by NaOH solution was apparently smaller: 14.5%, 13.7%, and 12.9%, respectively, for membranes M10, M20, and M30.

### 3.4. Dynamic Filtration Results

**Figure 8:**
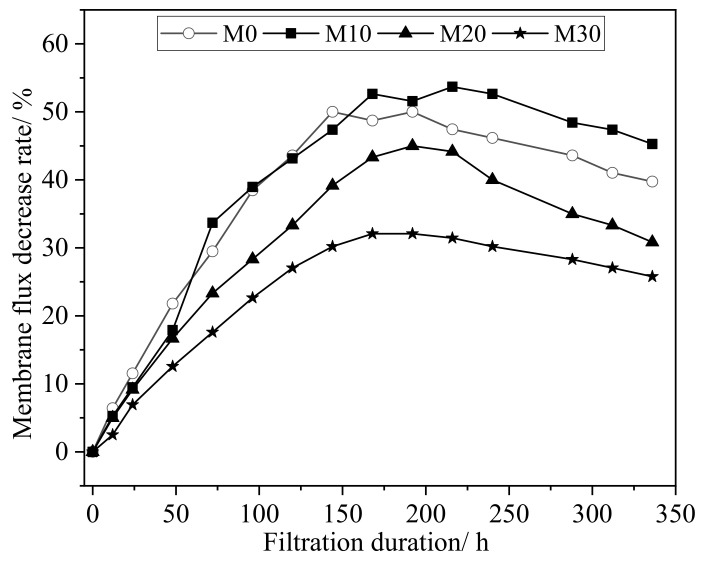
The rate of decrease in the membrane flux for the prepared membranes when subjected to dynamic continuous filtration with H_2_SO_4_ solution at an H^+^ concentration of 0.01 mol·L^−1^ (transmembrane pressure: 0.1 MPa).

The initial results of the static immersion test conducted in corrosive solutions may only partially reflect the resistance performance of the prepared membranes. For example, in the actual filtration process, the membranes will be subjected to liquid flowing through their pore structures under pressure. The modified membrane was, therefore, subject to a continuous filtration test with H_2_SO_4_ solution at an H^+^ concentration of 0.01 mol·L^−1^ for as long as 336 h (14 days) and a transmembrane pressure of 0.1 MPa. The membrane water fluxes were measured at various times, and the corresponding rate of change in the water flux (relative to the initial water flux) was calculated, as shown in Figure 8. It was found that, generally, the rate of decrease in membrane flux increased as the filtration time increased for the first 168 h, and then it tended to gradually decrease, the latter of which may be due to the corrosion of the membrane by acid, leading to swelling of the PVDF polymer and reduction of the membrane pores. As can be observed in Figure 8, the modified membrane M30 showed the lowest rate of decrease in the membrane flux, which indicates that it has much better anticorrosion resistance than the pure PVDF membrane, M0.

## 4. Conclusions

PVDF/PTFE composite membranes with enhanced anticorrosion performance were obtained by a simple method involving the blending of PTFE nanoparticles with PVDF polymer and the fabrication of membranes through the common non-solvent-induced phase separation process. In contrast to the PTFE microparticles used in previous studies, the present study used PTFE nanoparticles. This greatly decreased the level of aggregation and resulted in a uniform dispersion in the membrane structures, factors that contributed to an improvement in the membrane’s anticorrosion property.

The prepared membranes were immersed in a few typical corrosive solutions, including H_2_SO_4_, HNO_3_, and HCl solutions with a high H^+^ concentration of 5 mol·L^−1^ (pH << 0) and NaOH solution with the 0.1 mol·L^−1^ OH^−^ (pH = 13), to investigate their anticorrosion performance. Although the breaking strength of all prepared membranes weakened as the immersion time increased from 1 to 6 months, the composite membranes with blended PTFE nanoparticles showed improved resistance to corrosion from these solutions, having a lower reduction in breaking strength than the pure PVDF membrane. After corrosion by the acidic solutions, the water flux of the membrane increased as the immersion time increased. While the M10, M20, and M30 membranes showed changes of 124.2%, 120.8%, and 113.2%, respectively, after being corroded in mol·L^−1^ H_2_SO_4_ solution for 6 months, the pure PVDF membrane, M0, showed a change of 107.7% after 3 months and then an increase to 150.0% after 6 months in the same H_2_SO_4_ solution. The surface roughness of the membranes can also be affected by corrosion by acidic or alkaline solutions. The effect on the average roughness was lowest with the HCl solution and greatest with the H_2_SO_4_ and HNO_3_ solutions. Since defluorination of the PVDF polymer may occur in alkaline solutions, the results of this study showed that the alkaline solution had a greater effect on the stability of the prepared membranes than the acidic solutions. However, the membranes blended with PTFE nanoparticles indeed had better corrosion resistance. During continuous filtration of the H_2_SO_4_ solution with an H^+^ concentration of 0.01 mol·L^−1^ for 336 h (14 days), the PVDF/PTFE composite membranes also demonstrated much better resistance and stability than the solo PVDF membrane. Hence, the simple method for preparing PVDF/PTFE composite membranes with a greatly enhanced anticorrosion performance presented in this study has great potential to expand the application of PVDF membranes for use in corrosive industrial effluent treatment.

## Figures and Tables

**Table 1:** Information on the different PVDF/PTFE composite membranes prepared in this study.

Composition or Physical Feature	Membrane Type			
	M0	M10	M20	M30
Weight of NMP/g	84	84	84	84
Weight of PVDF/g	16	16	16	16
Weight of 62% PTFE nanoemulsion/g	0	2.87	6.45	11.06
PTFE content relative to PVDF and PTFE/wt%	0	10	20	30
Total solid content in the casting solution/%	16	17.3	18.8	20.6
Breaking strength/MPa	4.16	3.74	3.25	2.97
Breaking elongation/%	246.8	217.2	175.2	131.0
WCA/°	78.6	81.2	83.8	88.5
Oil contact angle in water/°	100.3	106.1	114.7	125.0
Porosity/%	73.9	78.2	78.9	80.3
Water flux/L·m^−2^·h^−1^	7.8	9.5	12.0	15.9

**Table 2:** Relative tensile strength and rate of retention in tensile strength for prepared membranes immersed in different acid or alkaline solutions.

		Breaking Strength/MPa					Breaking Strength Retention Rate/%			
Immersion Solution		0	30	60	90	180	30	60	90	180
H_2_SO_4_	M0	4.16	3.51	3.06	2.80	2.32	84.3	73.6	67.3	55.8
	M10	3.74	3.24	2.89	2.62	2.23	86.6	77.3	70.1	59.6
	M20	3.25	2.85	2.52	2.33	2.08	87.7	77.5	71.7	64.0
	M30	2.97	2.68	2.41	2.26	2.07	90.2	81.1	76.1	69.7
HCl	M0	4.16	3.65	3.31	3.13	2.93	87.7	79.6	75.2	70.4
	M10	3.74	3.31	3.02	2.86	2.72	88.5	80.7	76.5	72.7
	M20	3.25	2.9	2.66	2.52	2.37	89.2	81.8	77.5	72.9
	M30	2.97	2.69	2.50	2.38	2.20	90.6	84.2	80.1	74.1
HNO_3_	M0	4.16	3.62	3.21	2.95	2.57	87.0	77.2	70.9	61.8
	M10	3.74	3.29	2.92	2.71	2.41	88.0	78.1	72.5	64.4
	M20	3.25	2.85	2.56	2.45	2.20	87.7	78.8	75.4	67.7
	M30	2.97	2.68	2.41	2.20	2.12	90.2	81.1	74.1	71.4
NaOH	M0	4.16	3.34	2.73	2.49	2.15	80.3	65.6	59.9	51.7
	M10	3.74	3.06	2.54	2.34	2.07	81.8	67.9	62.6	55.3
	M20	3.25	2.70	2.34	2.12	1.88	83.1	72.0	65.2	57.8
	M30	2.97	2.58	2.22	2.09	1.88	86.9	74.7	70.4	63.3

**Table 3:** Water flux of membranes after immersion in corrosive solutions for different periods of time.

		Flux/L/m^2^h					Increase in Flux/%			
Immersion Immersion Time/Days		0	30	60	90	180	30	60	90	180
H_2_SO_4_	M0	7.8	11.3	13.3	16.2	19.5	44.9	70.59	107.7	150.0
	M10	9.5	13.8	16.8	18.6	21.3	45.3	76.8	95.8	124.2
	M20	12.0	16.6	21.2	22.1	26.5	38.3	76.7	84.2	120.8
	M30	15.9	21.4	25.6	28.1	33.9	34.6	61.0	76.7	113.2
HCl	M0	7.8	9.9	11.8	13.1	14.6	26.9	51.3	67.9	87.2
	M10	9.5	14.2	14.8	16.1	18.3	49.5	55.8	69.5	92.65
	M20	12.0	15.4	17.9	21.2	21.3	28.3	49.2	76.7	77.5
	M30	15.9	20.1	23.2	25.0	28.1	26.4	45.9	57.2	76.7
HNO_3_	M0	7.8	10.3	12.2	13.3	14.2	32.1	56.4	70.5	82.1
	M10	9.5	13.2	15.4	17.0	19.5	38.9	62.1	78.9	105.3
	M20	12.0	15.8	17.5	20.1	22.8	31.7	45.8	67.5	90.0
	M30	15.9	21.1	24.9	27.1	29.6	32.7	56.6	70.4	86.2
NaOH	M0	7.8	9.5	11.1	11.5	12.8	21.8	42.3	47.4	64.1
	M10	9.5	12.0	13.7	14.7	16.6	26.3	44.2	54.7	74.7
	M20	12.0	14.5	16.4	17.5	17.6	20.8	36.7	45.8	46.7
	M30	15.9	19.2	21.7	23.3	25.2	20.8	36.5	46.5	58.5

**Table 4:** Average surface roughness of the prepared membranes. R.

	M0	M0_S6_	M30	M30_S6_	M30_N6_	M30_Cl6_	M30_Na6_
Ra (nm)	15.7	22.5	17.6	21.0	21.2	18.5	22.0
Rq (nm)	19.8	28.0	22.9	26.9	30.3	25.9	30.1

Ra: Arithmetic average of the surface roughness; Rq: Root-mean-square average of the surface roughness.

**Table 5:** The WCAs of the prepared membranes before and after immersion in different acidic and alkaline solutions for 180 days.

	Initial WCA/°	WCA after Immersion in Different Corrosive Solutions for 180 Days/°			
		H_2_SO_4_	HCl	HNO_3_	NaOH
M0	78.6	71.3	74.9	73.5	66.6
M10	81.2	73.1	76.5	75.7	69.4
M20	83.8	76.7	79.7	78.7	72.3
M30	88.5	81.4	84.2	83.7	77.1

## Data Availability

Not applicable.
